# Unseen Threats: The Long‐term Impact of PET‐Microplastics on Development of Male Reproductive Over a Lifetime

**DOI:** 10.1002/advs.202407585

**Published:** 2025-01-13

**Authors:** Seungjin Jeong, GyuDae Lee, Surye Park, Myeongjoo Son, Seungjun Lee, Bomi Ryu

**Affiliations:** ^1^ Department of Food Science and Nutrition Pukyong National University Busan 48513 Republic of Korea; ^2^ Department of Smart Green Technology Engineering Pukyong National University Busan 48513 Republic of Korea; ^3^ Department of Applied Biosciences Kyungpook National University Daegu 41566 Republic of Korea; ^4^ Department of Anatomy & Cell Biology, School of Medicine Kangwon National University Chuncheon 24341 Republic of Korea

**Keywords:** epididymis, polyethylene terephthalate, spermatogenesis, testis, transcriptome

## Abstract

The physical abrasion of plastics from simple everyday entered the food chain, with associated risks recently emphasized. Although many studies have reported the adverse effects of microplastics (MPs) on human, the reproductive implications of continuous exposure to physically abraded polyethylene terephthalate (PET)‐MPs remain unexplored. Ingestion of physically abraded PET‐MPs (size range: 50–100 µm) in mice from 5 to 34 weeks of age at an annual intake relevant dose of MPs (5 mg week^−1^) significantly impaired male reproductive function. Reductions in seminiferous tubule diameter and epithelial height are observed (*p* < 0.0001), with 32.2% decrease in Leydig cells and 24.3% reduction in testosterone levels (*p* < 0.05). The epididymis shows marked deterioration in all regions, with total sperm concentration significantly reduce from 17.0 × 10⁶ to 5.3 × 10⁶ (*p* < 0.01) and decrease motility. Transcriptome analysis demonstrates downregulation of genes related with gonadotropin‐releasing hormone secretion, testosterone biosynthesis, and Meiosin gene, which is for crucial spermatogenesis. Continuous ingestion of physically abraded PET‐MPs from plastic bottles adversely affected testicular and epididymal functions, leading to hormonal imbalances and abnormal sperm production. These findings raise concerns about the impact of commonly used plastics on male reproductive development, highlighting potential risks for future generations.

## Graphical abstract

This study explores the effects of long‐term ingestion of physically abraded microplastics derived from plastic water bottles on reproductive health, affecting sperm formation, maturation, and hormonal balance. Continuous ingestion of microplastics disrupts testicular structure and key genes involve in spermatogenesis, highlighting potential risks to male sexual development and growth.

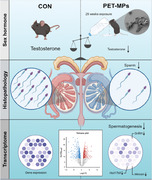

## 1 Introduction

The world is dominated by plastic. Plastic is cheap, versatile, and used extensively in various industries, from food packaging to medical devices, transportation, and construction. However, the excessive use of plastic materials has led to plastic pollution issues, particularly the urgent problem of MPs. A previous study demonstrated that MPs (≤ 5 mm) can be generated through physical abrasion, mechanical degradation, chemical degradation, and UV degradation.^[^ ^1^ ^]^ Ultimately, these MPs persistently infiltrate the environment and food, consequently entering the human body unintentionally via multiple routes.^[^ ^2^ ^]^

MPs exist as primary MPs (e.g., virgin plastic pellets and microbeads), which are directly produced as fine particles, and secondary MPs (e.g., degraded bottles and fragmented packaging), which result from the fragmentation of larger plastic debris. Especially, the creation of secondary MPs can occur even during simple actions like opening a plastic bottle cap.^[^ ^2^, ^3^, ^4^, ^5^ ^]^ A study has also indicated that tire wear particles due to mechanical abrasion are a primary source of MPs in the environment. Meanwhile, other research found that a disposable 1‐liter water bottle, which is primarily made from PET, contains 240,000 plastic particles, demonstrating the potential effects on humans through ingestion.^[^ ^6^, ^7^ ^]^ Physically abraded MPs can easily enter the food chain through various pathways, posing a threat to human health.

Studies suggest that humans can intake up to 250 grams of MPs annually.^[^ ^8^ ^]^ Vulnerable populations, such as pregnant women and neonatal infants, are also exposed to MPs. In particular, MPs smaller than 10 µm can penetrate cell membranes, thereby affecting the circulatory system and posing unknown risks to human health, indicating that even newborns cannot escape ingestion of MPs.^[^ ^9^ ^]^ Children show a higher intake rate of MPs relative to their body weight compared to adults.^[^ ^10^ ^]^ This raises significant concerns that the ingestion of MPs by infants and children could continue into adulthood, causing adverse effects throughout their entire lifecycle, including growth and secondary growth phases. MPs act as carriers for environmental pollutants and physical stressors, posing various risks to humans, including hormonal imbalances, incomplete growth, endocrine disruptions, and reproductive issues.^[^ ^11^, ^12^, ^13^, ^14^, ^15^, ^16^ ^]^

In recent years, a study on the relationship between MPs intake and reproductive functions has been actively investigated. Remarkably, MPs were detected in the testes, the human male reproductive organ, which has the heightened focus on the potential effects of MPs on male growth, maturation, and reproductive system.^[^ ^17^, ^18^ ^]^ In Deng et al.’s study (2022), polystyrene (PS)‐MPs, the most widely used thermoplastic resin for foam insulation and disposable or single‐use food packaging, were administered to 5‐week‐old mice for 21 weeks. The result revealed a decline in sperm quality and negative effects on the reproductive capabilities of male mice, highlighting the transgenerational impacts of MPs ingestion.^[^ ^19^ ^]^

PET accounts for 7.2% of the total annual plastic production and is a major component of water bottles and food packaging, which are potential ingestion routes for MPs.^[^ ^20^ ^]^ In children aged 0–5 years in the United States, levels of PET detected in feces were significantly higher than those of polycarbonate, which is used for food containers such as baby bottles. This finding highlights that infants and children are at risk of long‐term PET‐MPs exposure from an early age.^[^ ^21^ ^]^ However, the physical risks associated with PET‐MPs accumulating in the body from the peripubertal period onward have not yet been studied. Ingestion of PET‐MPs derived from water bottles during the critical maturing periods from adolescence to adulthood period may pose risks to maturity. Therefore, further research efforts are needed to ensure healthy maturity and development from adolescence to adulthood.

Previous studies contain such limitations as short‐term exposure to excessive amounts of MPs in the form of uniform‐sized beads instead of the irregular forms of MPs commonly found in the environment. Therefore, the present study hypothesized that long‐term ingestion (29 weeks) of physically abraded PET‐MPs at annual intake‐relevant doses (5 mg/week) from peripubertal to adult period could affect testicular and epididymal development and maturation. To date, this is the first in vivo evidence in terrestrial mammals demonstrating that continuous ingestion of physically abraded PET‐MPs from peripubertal to adult period adversely affects testicular growth and maturation, correlating with male reproductive development and overall growth. The study highlights the potential risks associated with long‐term exposure to MPs and warrants further research on its implications for human health.

## 2 Results

### 2.1 Characteristics of Physically Abraded PET‐MPs from Water Bottles

**Figure 1:**
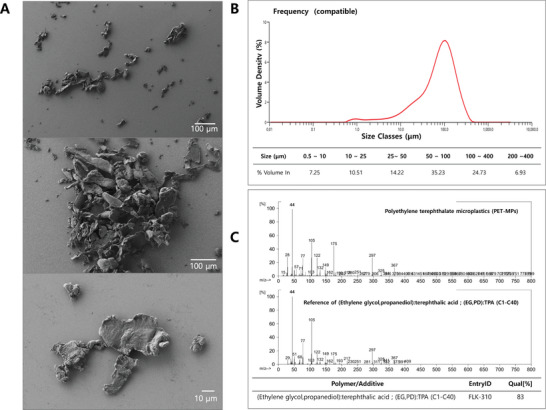
Characteristics of Physically abraded PET‐MPs from Water Bottles. A) Scanning electron microscopy (SEM) images of PET‐MPs at different magnifications. B) Particle size distribution of PET‐MPs (volume density, %). The table below the graph details the percentage volume of PET‐MPs within specific size ranges: 0.5‐10 µm (7.25%), 10–25 µm (10.51%), 25–50 µm (14.22%), 50–100 µm (35.23%), 100–200 µm (24.73%), and 200–400 µm (6.93%). C) Pyrolysis‐gas chromatography/mass spectrometry (Py‐GC/MS) analysis of PET‐MPs. The upper chromatogram shows the characteristic peaks of PET‐MPs. The lower chromatogram serves as a reference of (Ethylene glycol, propanediol): terephthalic acid (EG, PD): TPA (C1‐C40). The qualitative identification of the polymer and additive is provided with a quality match percentage of 83% in F‐search software.

PET‐MPs were generated through mechanical grinding using water bottleas the source material. Major characteristics (e.g., shape, size distribution, and chemical composition) of the generated PET‐MPs were analyzed (**Figure** **1**). The morphology of generated PET‐MPs was characterized using scanning electron microscopy (SEM) (Figure 1A) and distribution of PET‐MPs’ particle size was measured using particle size analyzer (Figure 1B). The SEM images showed the rough and irregular shape and fragments as the PET‐MPs abraded using water bottles. In addition, the Figure 1B shows the volume density of different size classes of generated PET‐MPs, with the majority of particles falling within the 50–100 µm range. The percentage volume of PET‐MPs within specific size ranges was as follows: 0.5‐10 µm (7.25%), 10–25 µm (10.51%), 25–50 µm (14.22%), 50–100 µm (35.23%), 100–200 µm (24.73%), and 200–400 µm (6.93%).

Using pyrolysis‐gas chromatography/mass spectrometry (Py‐GC/MS) analysis, PET‐MPs in this study were identified by quantifying terephthalic acid (TPA) in PET (Figure 1C), and the plastic water bottle was identified as PET containing 92.58% of TPA, as 0.11 mg of TPA per 0.12 mg of a water bottle (Figure S1, Supporting Information). The mass spectra of PET‐MPs were also implemented with the reference of ethylene glycol, propanediol (EG, PD): TPA(C1‐C40) polymers in F‐search software. For the characteristic of PET‐MPs, the pyrolysis profile of PET‐MPs analyzed by Py‐GC/MS features 41 products along with a set of m/z signals corresponding to PET in NIST (National Institute of standards and technology Library) (Table S1, Supporting Information) and is also addressed benzoic acid as indicator component of PET‐MPs (Figure S2, Supporting Information).^[^ ^22^ ^]^

### 2.2 Impact of Long‐term PET‐MPs Exposure from Peripubertal to Adult Period on Seminiferous Tubule Structure, Testicular Development, Testosterone Production, and Spermatogenesis

**Figure 2:**
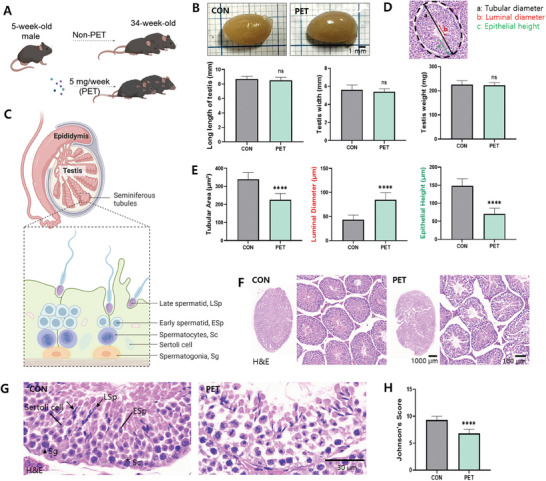
Effects of ingesting PET‐MPs on testicular growth in mice from peripubertal to adult period. A) Experimental design for assessing the impact of PET‐MPs. Five‐week‐old male mice were administered PET‐MPs through feed at a dose of 5 mg week^−1^ until 34 weeks of age. B) Gross morphology of testis from mice in CON and PET. The images depict the appearance of testis from both groups, and the graphs show measurements of testis length (left), width (middle), and weight (right). C) Schematic diagram of the male reproductive system, highlighting the testis and seminiferous tubules. The inset shows detailed cellular components including late spermatid, early spermatid, spermatocytes, Sertoli cells, and spermatogonia. D) Histological section of a seminiferous tubule showing measurements for tubular diameter (a), luminal diameter (b), and epithelial height (c). E) Quantitative analysis of tubular area (µm^2^), luminal diameter (µm), and epithelial height (µm) in seminiferous tubules from CON and PET represented through a bar chart. F) Hematoxylin and eosin (H&E) stained sections of seminiferous tubules from mice in CON and PET. G) High magnification H&E‐stained sections showing detailed cellular architecture of seminiferous tubules in mice from CON and PET. The images highlight the arrangement and appearance of Sertoli cells, spermatocytes, spermatogonia, early spermatids, and late spermatids. H) Johnsen's score for seminiferous tubule morphology in CON and PET. The data are presented as the means ± SDs. ****p < 0.0001; n.s., *p* > 0.05. Statistical analysis in panels B, C, and E was performed by unpaired Student's *t*‐test.

Using male C57BL/6N mice, the reproductive toxicity effects of long‐term PET‐MPs ingestion (5 mg week^−1^) from 5 to 34 weeks of age, corresponding to the period from peripubertal to adult period, were evaluated (**Figure** **2**A).

Body weight significantly increased in PET‐MPs ingested mice over 29 weeks (*p* < 0.05) (Figure S3A, Supporting Information; *p* < 0.05). However, no statistical differences were observed in body length (Figure S3B, Supporting Information), testicular weight, or testicular size (length and width) (Figure 2B).

Within the testis, the seminiferous tubules contain spermatogonia, spermatocytes, and spermatids (Figure 2C). PET‐MPs ingestion resulted in decreased tubular area (*p* < 0.0001), tubular diameter (TD; *p* < 0.0001), and epithelial height (EH; *p* < 0.0001) of the seminiferous tubules (Figure 2D,E). The alterations observed in TD and EH within the seminiferous tubules correlate with pathological differences characterized by irregular and structurally unstable seminiferous tubules in the testicular tissue (Figure 2F). Higher magnification analysis of the seminiferous tubules showed distinct components: spermatogonia with round, darkly stained nuclei at the periphery; spermatocytes with large, round nuclei above the spermatogonia; late spermatids with dark blue heads and eosinophilic flagella; and elongated Sertoli cells with pale nuclei and prominent nucleoli (Figure 2G). In the PET‐MPs group (PET), these tubules displayed disorganized Sertoli cells, reduced spermatogonia, and fewer mature spermatozoa compared to the control group (CON) (Figure 2G). This observation was further corroborated by the significantly reduced Johnson score of mice in the PET, a semi‐quantitative scoring system used to evaluate spermatogenic activity based on the presence and development of various germ cell types within the seminiferous tubules (Figure 2H) (details on the scoring criteria can be found in Table S2, Supporting Information.)

**Figure 3:**
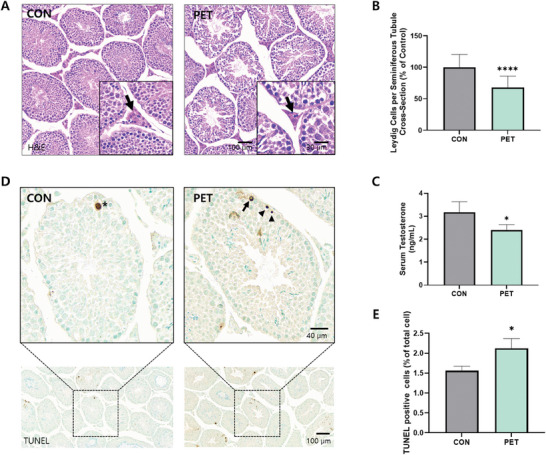
Impact of PET‐MPs on Leydig Cells, testosterone levels, and cell apoptosis in mice. A) H&E‐stained sections of seminiferous tubules from CON and PET, showing Leydig cells (indicated by arrows). Insets provide a higher magnification view of Leydig cells within the interstitial spaces. B) Quantification of Leydig cells per seminiferous tubule cross‐section (expressed as a percentage of control). C) Testosterone levels (ng/mL) in serum of mice from CON and PET. D) Immunohistochemical staining for TUNEL‐positive cells in seminiferous tubules from CON and PET. Insets provide higher magnification images, indicating the presence of apoptotic cells (arrows). E) Quantification of TUNEL‐positive cells as a percentage of the total cell population in seminiferous tubules. The data are presented as the means ± SDs. *p < 0.05; ****p < 0.0001. Statistical analysis in panels B, C, and E was performed by unpaired Student's *t*‐test.

Histological analysis revealed reduced Leydig cell populations in the interstitial space between seminiferous tubules of the PET‐MPs ingested mice (**Figure** **3**A). The PET shows a significant 32.16% reduction in Leydig cell count compared to the CON (Figure 3B; *p* < 0.0001). The mice of the PET significantly decreased testosterone levels in serum (Figure 3C; *p* < 0.05). Levels of testosterone in serum showed a significant 24.34% decrease compared to the CON, which showed levels of approximately 3 ng mL^−1^. Figure 3D,E revealed increased apoptosis of various cell types, including spermatogonia, spermatocytes, and spermatids in the PET (*p* < 0.05). Quantification of TdT‐mediated dUTP‐biotin nick‐end labeling (TUNEL)‐positive cells as a percentage of the total cell population in seminiferous tubules. PET‐MPs ingestion significantly increased the number of apoptotic cells (*p* < 0.05).

### 2.3 Impact of PET‐MPs on Sperm Maturation of Epididymis

**Figure 4:**
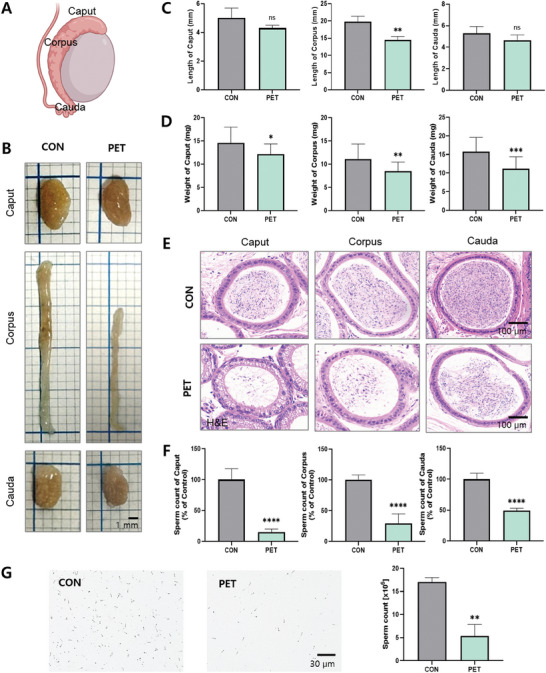
Impact of PET‐MPs on sperm maturation of epididymis. A) Schematic diagram of the epididymis, highlighting its three main regions: caput, corpus, and cauda. B) Gross morphology of the caput, corpus, and cauda regions of the epididymis from CON and PET. Images show the physical appearance of each region, highlighting differences between the two groups. C) Length measurements (mm) of the caput, corpus, and cauda regions of the epididymis in CON and PET. D) Weight measurements (mg) of the caput, corpus, and cauda regions of the epididymis in CON and PET. E) H&E‐stained sections of the caput, corpus, and cauda regions of the epididymis from CON and PET. The images show the structural differences in the epididymal regions between the two groups. F) Sperm count (expressed as a percentage of control) in the caput, corpus, and cauda regions of the epididymis in CON and PET. G) Representative microscopic images (left and center) of sperm samples obtained from the cauda epididymis of CON and PET, Scale bar: 30 µm. Quantitative analysis of sperm count (right) represented through a bar chart. The data are presented as the means ± SDs. *p < 0.05; **p < 0.01; ***p < 0.001; ****p < 0.0001; n.s., *p* > 0.05. Statistical analysis in panels C, D, F, and G was performed by unpaired Student's *t*‐test.

The epididymis, an organ where sperm mature and are stored after being produced in the testes, is divided into three sections: the caput, corpus, and cauda (**Figure** **4**A).^[^ ^23^ ^]^ The mice ingested with PET‐MPs from peripubertal to adult period were found to result in a significant 26.89% reduction in corpus length compared to the CON (*p* < 0.01), with no significant changes observed in the lengths of the caput and cauda (Figure 4B,C; *p* < 0.01). A significant reduction in the weight of the epididymis was observed in the PET, affecting the caput (*p* < 0.05), corpus (*p* < 0.01), and cauda (*p* < 0.001) (Figure 4D). The H&E staining results of the epididymal tissue confirmed that the number of sperm in the tubules of the caput, corpus, and cauda of the epididymis was significantly decreased (Figure 4E,F; *p* < 0.0001). Ingestion of PET‐MPs significantly reduced number of sperm in all regions of the epididymis, including caput, corpus, and cauda showing reductions of 84.81%, 70.76%, and 50.95%, respectively (Figure 4F; *p* < 0.0001).

Sperm counts obtained from the cauda epididymis showed a significant decrease in sperm density in the PET compared to the CON. The CON had a sperm count of 17.0 × 10⁶, whereas the PET had a significantly lower count of 5.3 × 10⁶ (Figure 4G; *p* < 0.01).^[^ ^24^, ^25^ ^]^ Additionally, sperm motility analysis demonstrated decreased sperm movement in the PET (Video S1‐S6, Supporting Information).

These results indicated that ingestion of PET‐MPs adversely affects the structure and function of the epididymis, leading to reduced length and weight of the epididymal regions and a significant decrease in sperm count.

### 2.4 Transcriptomic Insights into Testosterone and Meiotic Gene Disruptions from Chronic PET‐MPs Ingestion

**Figure 5:**
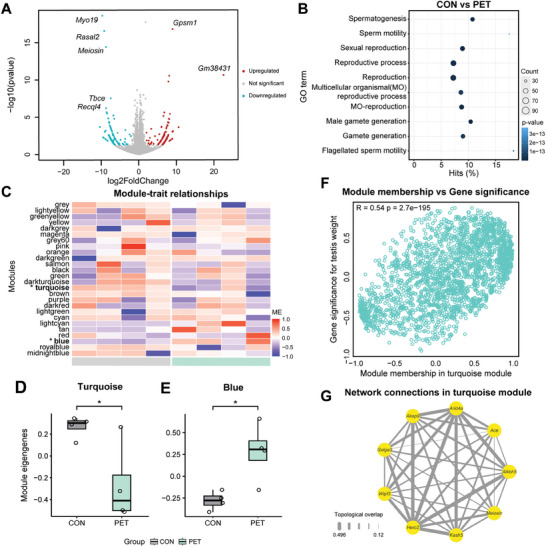
Variation in testicular gene expression patterns with PET‐MPs ingestion and their correlation with testicular weight. A) Volcano plot showing DEG in testes from PET‐MPs ingested mice compared to the controls (padj < 0.05, |log_2_FC| >2). Genes with significant up‐regulation are shown in red, down‐regulation in blue, and non‐significant changes in grey. Key genes with notable changes are labeled. B) GO term enrichment analysis of DEG. The bubble plot shows enriched GO terms related to reproduction, with bubble size representing the number of genes and color indicating p‐value significance. C) Heatmap showing the eigengene values of the clustered modules for each sample. The colors on the y‐axis represent the different modules, and modules showing significant differences between groups (turquoise, blue; *p < 0.05) are marked with an asterisk. D) Box plot of module eigengene values for the turquoise module in CON and PET. Ingestion of PET‐MPs significantly altered the eigengene values in this module (*p < 0.05; Mann‐Whitney test). E) Box plot of module eigengene values for the blue module in CON and PET. Ingestion of PET‐MPs significantly altered the eigengene values in this module (*p < 0.05; Mann‐Whitney test). F) Scatter plot showing the correlation between module membership and gene significance for testes weight in the turquoise module. The plot shows a strong positive correlation (R = 0.54, p = 2.7e–195). G) The network connection of genes belonging to the turquoise module that are involved in the spermatogenesis GO term. A total of eight genes are connected by grey lines of different thicknesses depending on the degree of topological overlap.

To investigate the mechanisms by which PET‐MPs affect the growth and maturation of the testes at the molecular level, RNA transcriptome data from the testes of mice that ingested PET‐MPs for 29 weeks were analyzed. By displaying differentially expressed genes (DEGs) in a volcano plot using DESeq2, a total of 138 upregulated and 131 downregulated genes were identified, highlighting those with significantly altered expression levels compared to the CON (**Figure** **5**A). Further analysis of Gene Ontology (GO) terms for all DEGs, rather than individual genes, revealed significant associations with developmental process involved in reproduction, including spermatogenesis and sperm motility (Figure 5B; padj<0.05).

Based on the overall transcriptional expression patterns, a co‐expression network was constructed to understand the association between gene expression patterns and phenotypes (testicular weight). Predict hierarchical clusters which is called to modules through dendrogram were generated (Figure S4, Supporting Information). Modules with similar expression patterns, represented by 26 color bars on the y‐axis of the heatmap (Figure 5C). The eigengene values of the turquoise and blue modules were significantly different compared to the CON (Figure 5D,E; *p* < 0.05).

Focusing on changes in testes weight relative to body weight, analysis was performed on the association between turquoise module, which was the most significant among the modules (P = 0.036; limma). The y‐axis represents the correlation between each gene in the transcriptome and testes weight relative to body weight per sample, while the x‐axis indicates module membership, showing the correlation between all genes and eigengene of turquoise module (Figure 5F). In the turquoise module, gene significance and module membership were significantly positively correlated (Figure 5G). These results are consistent with the pattern of turquoise module eigengene in the PET and suggest an association with reduced testicular weight.

The overlapping genes belonging to the GO terms that were significant among the genes in the turquoise module were shown in the network plot (Figure 5G). The *Meiosin* gene was also included as shown in Table S3, Supporting Information.

**Figure 6:**
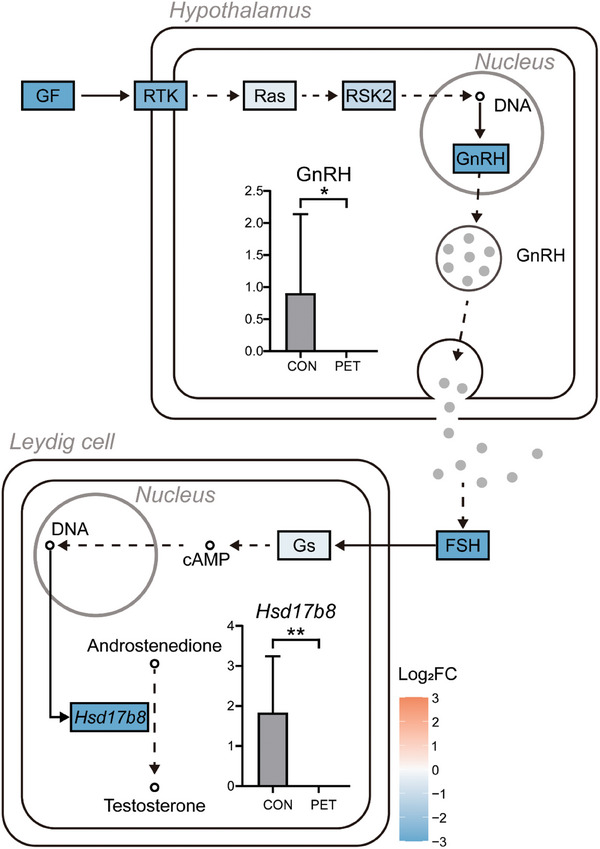
Inhibition of testosterone production due to ingestion of PET‐MPs. Summarization of GnRH secretion and testosterone biosynthesis pathway through KEGG analysis. GF: vascular endothelial growth factor A. RTK: colony stimulating factor 1 receptor. Ras: related RAS viral (r‐ras) concogene. RSK2: ribosomal protein S6 kinase polypeptide 3. GnRH: gonadotropin releasing hormone 1. FSH: secreted phosphoprotein 1. Gs: guanine nucleotide‐binding protein G(s) subunit alpha. Hsd17b8: 17beta‐estradiol 17‐dehydrogenase. GnRH and *Hsd17b8* gene represented by box plots indicate statistically significant differences (*p < 0.05; **p < 0.01 Mann‐Whitney test).

KEGG pathway analysis also revealed significant gene changes associated with gonadotropin‐releasing hormone (GnRH) secretion and testosterone biosynthesis pathway (**Figure** **6**).

These results indicate that ingestion of PET‐MPs leads to significant changes in gene expression associated with reproductive processes and testicular function, affecting key gene networks and modules.

## 3 Discussion

Numerous previous studies have focused on the detection of MPs generated through weathering or environmental degradation in various organs and their subsequent short‐term effects on animal and/or human health.^[^ ^26^ ^]^ To evaluate the effects of MPs on animal and/or human health, previous studies used the commercial MPs, which are bead‐shaped and have a smooth surface and regular morphology. However, MPs commonly found in the environment, as well as those directly exposed to physical abrasion during plastic use, differ in shape and distribution of particle size from commercial bead‐shaped MPs generally used in previous studies.^[^ ^27^ ^]^

While previous studies have provided valuable insights into environmental exposure to MPs, they may not fully represent the MPs encountered in daily life. MPs generated from physical force for the use of disposable goods are directly ingested.^[^ ^28^ ^]^ Specifically, a recent study reported that 240,000 plastic particles were detected in disposable 1‐liter water bottles.^[^ ^7^ ^]^ Despite ongoing exposure to plastic particles through wear of commonly used plastics, the direct risks associated with ingestion of these particles remain unknown. Therefore, the findings demonstrated that PET‐MPs generated through mechanical abrasion of water bottles exhibited rough and irregular surface morphology, distinct from the smooth surfaces typically observed in commercially generated MPs, consistent with previous reports.^[^ ^29^ ^]^

The human body is exposed to MPs through various pathways, such as ingestion, inhalation, and dermal contact. Therefore, studies are being conducted on the health effects of exposure to MPs, including inflammation and disruption of the nervous and immune systems.^[^ ^30^ ^]^ Unnoticed ingestion of MPs is of particular concern due to its impacts on human maturation and development.^[^ ^31^ ^]^ A study from 2024 reported detection of a significant amount of MPs in sperm from males aged 10.^[^ ^32^ ^]^ This result indicates reproductive function impairment due to the impact of MPs ingestion on sperm. MPs have been continuously detected in the environment, including in water, soil, and air, and in various foods. Consequently, studies are needed to investigate the effects of long‐term MPs ingestion on males, especially development of reproductivity, from infancy through adulthood. Therefore, this study highlights long‐term exposure of mice to PET‐MPs from peripubertal to adulthood period to evaluate effects on growth and reproduction.

The outer surface of the seminiferous tubules contains the spermatogonia, from which the tightly regulated process of spermatogenesis begins.^[^ ^33^ ^]^ These stem cell‐derived cells undergo mitosis in the spermatocyte stage as they move towards the center, where they differentiate into spermatozoa under the influence of Sertoli cells.^[^ ^34^ ^]^ In this study, histological analysis revealed structural changes in the seminiferous tubules, particularly in TD and EH measurements. These changes are particularly significant because the seminiferous tubular environment is crucial for proper spermatogenesis.^[^ ^35^ ^]^ The length of TD represents the overall diameter of the seminiferous tubule. This space has an important influence on the differentiation and maturation of spermatids in that it facilitates an environment that provides nutrients and hormones necessary for sperm cell differentiation.^[^ ^36^ ^]^ EH is the area where sperm cell growth and differentiation occur within the seminiferous tubule. By maintaining appropriate cell density, Sertoli cells smoothly support sperm cell differentiation and seminiferous tubule formation.^[^ ^33^, ^37^, ^38^ ^]^ The fact that the lengths of TD and EH were shortened following PET‐MPs ingestion suggests that PET‐MPs suppress the differentiation and growth of sperm cells.

Inside the tubules, two main cell types are present: 1) germ cells, which develop into sperm, and 2) Sertoli cells, which support the germ cells. Spermatogonia, located at the periphery, have round, darkly stained nuclei due to hematoxylin. Spermatocytes, found above the spermatogonia, possess large, round nuclei with distinct chromatin patterns. Late spermatids are identifiable by their dark blue heads and thread‐like, pink‐stained flagella (eosinophilic) extending towards the lumen. Sertoli cells are elongated, with pale nuclei and a prominent nucleolus, and their cytoplasm extends from the tubule periphery towards the lumen. The observed disorganization of this precise cellular architecture in mice that have ingested PET‐MPs, particularly the disruption of Sertoli cells and reduced germ cell populations, suggests significant impairment of the spermatogenic process. The presence of limited late spermatids, combined with reduced Johnson scores, indicates a fundamental disruption in the progression of spermatogenesis, likely affecting multiple stages of sperm cell development and maturation.

Most studies looked at short‐term, high doses of MPs and their harm to mouse testicles.^[^ ^39^, ^40^, ^41^ ^]^ In this study, continuous ingestion of physically abraded PET‐MPs from peripubertal to adult period, at doses equivalent to annual plastic intake, resulted in serious structural and functional disruption within the testes. These results highlight the adverse effects on testicular growth and maturation and indicate the broader testicular risks associated with ingestion of PET‐MPs.

In addition to these tubular structures, the interstitial cell population is located as a mall cluster and lies in the space between adjoining seminiferous tubules. They consist mostly of Leydig cells, the main source of testosterone in the male. Luteinizing hormone directly stimulates Leydig cells to produce testosterone, while FSH supports this process indirectly through its effect on Sertoli cells.^[^ ^39^, ^40^, ^41^, ^42^ ^]^ Therefore, both hormones are essential for the proper functioning of Leydig cells and the maintenance of male reproductive health.^[^ ^43^ ^]^ Impairment or reduction in Leydig cell function leads to decreased testosterone production, resulting in lower blood testosterone levels.^[^ ^44^ ^]^ Testosterone contributes to spermatogenesis, differentiation of spermatogonia, and reproductive functions in males.^[^ ^45^, ^46^ ^]^

The disruption of hormonal balance by PET‐MPs ingestion has significant implications for male reproductive function. The reduction of Leydig cells observed in mice following long‐term ingestion of PET‐MPs suggests a direct impact on the testosterone production mechanism. This effect is particularly noteworthy as testosterone levels in C57BL/6 mice are typically maintained at specific levels for proper reproductive function.^[^ ^47^ ^]^ The chronic exposure to PET‐MPs appears to interfere with this homeostasis through multiple mechanisms, including increased apoptosis of various testicular cell types.^[^ ^48^, ^49^ ^]^ This increased cell death, particularly in spermatogenic cells, indicates a comprehensive disruption of the reproductive system. Furthermore, the relationship between reduced Leydig cell numbers, decreased testosterone production, and increased apoptosis suggests a cascade of reproductive impairments.^[^ ^50^, ^51^ ^]^ PET‐MPs ingestion appears to initiate a cycle where reduced testosterone levels affect Sertoli cell function, which in turn compromises spermatogonial survival and development. This interconnected disruption of the testicular microenvironment demonstrates how PET‐MPs exposure can affect multiple aspects of male reproductive function simultaneously, from hormone production to germ cell development and maturation.

In this study, the detrimental effects of long‐term ingestion of PET‐MPs on spermatogenesis and maturation within the testes were observed (Figures 2, 3). Although the differentiation and maturation of sperm in the testes are immature due to ingestion of PET‐MPs, the sperm formed in the testes are further matured by the epididymal fluid as they pass through the epididymal duct and acquire motility and fertilization ability.^[^ ^52^ ^]^ The caput of the epididymis is where sperm first enter, where sperm maturation begins and the sperm are concentrated and stored for a while before moving to the corpus.^[^ ^53^ ^]^ In the corpus of the epididymis, as the period of exposure of sperm to epididymal fluid increases, sperm maturation becomes active and sperm begin to acquire motility and fertilization ability.^[^ ^54^ ^]^ The cauda of the epididymis is where sperm are highly concentrated and stored.^[^ ^55^ ^]^ In cauda, ​​the sperm reach full maturity and full fertilizing capacity before being released through the vas deferens.^[^ ^56^ ^]^ The transit time for spermatozoa generated in the testes to pass through the epididymis is approximately 10 days.^[^ ^57^ ^]^ This duration is based on the assumption that the epididymis is of normal length. Ingestion of PET‐MPs shortens the length of the corpus, thereby reducing the transit time through the epididymis. This reduced transit time decreases the duration available for sperm maturation and the acquisition of motility. This indicates that the ingestion of abraded PET‐MPs not only negatively impacts the differentiation and maturation of spermatozoa in the testes but also adversely affects the maturation processes occurring within the epididymis. Consequently, this indicates that the unnoticed ingestion of abraded PET‐MPs in daily life from the peripubertal period through adulthood may have harmful effects on sperm maturation and development.^[^ ^58^ ^]^ Furthermore, the findings of this study verify the reproductive toxicity caused by long‐term ingestion of MPs, suggesting that it may also impact the ability to produce next generations.

At the molecular level, transcriptomic analysis in this study revealed significant insights into reproductive dysfunction mediated by PET‐MPs (Figures 5, 6). Especially, the *Meiosin* gene, which is significantly down‐regulated in the PET‐MPs group (PET) plays a crucial role as a meiosis initiator for the transition from mitosis to meiosis, while decreased expression of the *Recql4* gene has been associated with reproductive impairment; this further supports the mechanistic link between exposure to PET‐MPs and reproductive dysfunction.^[^ ^59^, ^60^ ^]^ These molecular changes align with previous studies on other microplastics, particularly PS, which demonstrated similar impairments in spermatogenesis‐related gene expression.^[^ ^61^, ^62^ ^]^ Notably, the transcriptomic analysis results revealed the potential risk of PET‐MPs with the ingestion of low dose of PET‐MPs on reproductive functions, particularly testicular function. The expression of testosterone, a hormone influencing testicular growth and maturation, is regulated by GnRH secreted from the hypothalamus and FSH from the pituitary gland.^[^ ^63^ ^]^ While these transcriptional changes suggest potential alterations in hormone‐related pathways, further studies measuring actual hormone levels (GnRH, FSH, and LH) would be needed to establish direct causal relationships between PET‐MPs exposure and endocrine disruption. The downregulation of hormone‐related genes observed in the transcriptome analysis corresponded with decreased Leydig cell numbers and reduced testosterone levels, though the precise molecular mechanisms require additional investigation.^[^ ^64^ ^]^

In summary, the reduced testes weight relative to body weight in mice following long‐term ingestion of physically abraded PET‐MPs from the peripubertal period to adulthood is attributed to inhibited GnRH secretion from the hypothalamus and subsequent reduction in testosterone hormones within the testes. These hormonal changes significantly impacted spermatogenesis and maturation, highlighting the detrimental effects of PET‐MPs ingestion on development of male reproductive function.

## 4 Conclusion

The study warns of the potential risks to human health from continuous ingestion of physically abraded PET‐MPs due to use of disposable plastic bottles in daily life. Specifically, long‐term ingestion from the peripubertal period to adulthood (i.e., 29 weeks) of physically abraded PET‐MPs at an annual intake relevant dose of MPs (5 mg week^−1^) in mice adversely impacted the testes and epididymis, suppressing sperm production and maturation while causing imbalance in male hormones.

(i) The findings emphasize that physically abraded PET‐MPs can enter the food chain undetected, accumulate in the body, and negatively impact reproductive health. This emphasizes the significant threat to human health posed by ingestion of even small amounts of MPs in daily life already pose a significant threat to human. Further study is needed to investigate the effects of long‐term ingestion of physically abraded PET‐MPs on the reproductive functions of mammals and their transgenerational impacts on offspring.
(ii) Ingestion of long‐term PET‐MPs led to significant alterations in seminiferous tubule morphology and spermatogenic cell development, suggesting impaired sperm production. Furthermore, chronic ingestion of PET‐MPs results in reduced Leydig cell count within the testes, leading to a substantial suppression of reproductive hormones in a mammalian model. Consequently, impaired differentiation of Sertoli cells prevents sperm formation. Moreover, the reduced sperm cells face challenges in achieving sufficient maturation within the shortened corpus region of the epididymis affected by PET‐MPs. This underscores the adverse impacts of PET‐MPs, ingested continuously and unknowingly in daily life, on testicular and epididymal health, potentially suppressing sperm production and maturation and causing an imbalance in male hormones.
(iii) Transcriptome profiling confirmed that ingestion of PET‐MPs altered the expression levels of various genes associated with spermatogenesis. Specifically, impaired expression patterns were observed in pathways related to testosterone biosynthesis through GnRH secretion. Furthermore, co‐expression network analysis identified clusters of reproduction‐related genes within the turquoise module that may be associated with reproductive parameters. This correlation is likely explained by the inclusion of numerous reproduction‐related genes within the turquoise module. The findings suggest that chronic MPs ingestion could potentially lead to hormonal imbalances and impaired reproductive function in males.

To address these concerns, immediate actions are needed, including policy implementations for reducing plastic usage, developing eco‐friendly alternatives, and improving public awareness of MP exposure risks. Furthermore, several crucial research challenges remain to be addressed by next‐generation researchers: (1) investigation of potential transgenerational effects of PET‐MP exposure on offspring reproductive health and (2) examination of impacts on female reproductive function, particularly during critical developmental periods. These research directions are essential for understanding the full scope of MP‐induced reproductive toxicity and protecting future generations from the increasing threat of environmental plastic contamination.

With male fertility increasingly threatened by various environmental pollutants, the research focuses on the unseen threats posed by small amounts of PET‐MPs that have already entered the food chain and been continuously ingested since the peripubertal period, while also warning of the future challenges children may face.

## 5 Experimental Section

### Analysis of PET‐MPs by Using Scanning Electron Microscopy (SEM) and Particle Size Analyzer

This study obtained PET‐MPs by grinding water bottles produced in Korea and filtering them through a mesh with a pore size of 500 µm or less. To select small‐sized plastics, stainless steel mesh was used to obtain MPs smaller than 500 µm.

One gram was taken only of particle sizes <500 µm on the pan to be measured via laser diffraction using a Malvern Panalytical particle size analyzer (model Mastersizer 3000) (Malvern, UK) in dry dispersion mode with an air pressure of 3 bar (gauge). The particle size analysis was performed in triplicate.

FIB‐SEM Serial Surface View Imaging was performed using a Zeiss Crossbeam 550 (Zeiss Microscopy GmbH, Oberkochen, Germany) dual beam microscope (FIB‐SEM). The samples of PET‐MPs were elevated to the height of 5 mm, which corresponds to the coincident point of the two beams and tilted to 54°. All SEM analyses were repeated three times independently.

### Ethical Approval

All animal experiments adhered to the National Guidelines for Animal Care and Use of Korea. The study received approval from the Southern Medical University Scientific Research Committee on Ethics in the Care and Use of Laboratory Animals (Permit No. PKNUIACUC‐2023‐22).

### Mouse Husbandry

A total of 33 four‐week‐old male C57BL/6 N mice, weighing 18–19 g, were utilized in this study. These mice were procured from Samtako Bio‐Korea Inc. in Korea. Upon arrival, a 7‐day acclimatization period was provided to allow the animals to adjust to the new environment. Housing conditions were maintained in a temperature‐controlled room at 25 ± 2 °C with a relative humidity of 50 ± 5% and a 12‐h light‐dark cycle was enforced (lights on from 7:00 a.m. to 7:00 p.m.). Mice were given access to food and sterilized water ad libitum. Random allocation into specific groups was performed based on their initial body weights at the beginning of the experiment. Mice were administered PET‐MPs through feed at doses of 5 mg per week for 29 weeks, during which body weight was measured weekly. The weekly dose of 5 mg PET‐MPs was determined by proportionally scaling down the reported human GARMI value (5 g/week) according to the average body weight ratio between humans and mice.^[^ ^15^ ^]^ Additionally, concentrations of PET‐MPs consumed per week were calculated based on amount of actual food consumption of mice (Figure S5, Supporting Information).

By measuring the length of mice from the tip of their nose to the base of their tail, the effect of PET‐MPs intake on the growth rate of mice was assessed.^[^ ^65^ ^]^

### Pyrolysis Gas Chromatography/Mass Spectrometry (Py‐GC/MS) Analysis of PET‐MPs

The MPs particles made PET was also detected with pyrolysis (EGA/Py‐3030D, Frontier lab) coupled to gas chromatography (8890 GC, Agilent Technologies) and mass spectrometry (5977B MSD, Agilent Technologies) (Py‐GC/MS). The analyses were conducted in triplicate to ensure reliability and reproducibility of the results.

The column dimensions were 30 m in length, 0.25 mm inner diameter, and 0.25 µm film thickness. The following temperature program was set to achieve chromatographic separation: held at 50 °C for 5 min, increased at 20 °C min^−1^ to 320 °C held for 38.5 min. Helium (purity 5.0) was used as the carrier gas with a vent flow of 1 mL min^−1^. Total ion current (TIC) mode was used to identify and quantify the polymers of target particles.

A comprehensive quality assurance and quality control (QA/QC) protocol was implemented throughout the entire experimental process, including sample collection, storage, processing, and analysis. All materials used during these processes were free of plastic contamination.

### Histological Morphometric Analysis

Paraffin‐embedded testes tissues were sectioned at 5 µm thickness. Tissues were fixed in 10% neutral buffered formalin (NBF) (HT501640, Sigma‐Aldrich Co., St. Louis, MO, USA). Deparaffinization was achieved by immersing the sections in xylene for 20 min followed by a graded ethanol series (100% to 70%, 3 min each). Subsequently, the slides were stained with hematoxylin (DAKO, UK) for 1 min and eosin (Sigma‐Aldrich) for 20 sec. Following three washes with water, the slides were mounted with a coverslip using a xylene‐based DPX mounting medium (Sigma‐Aldrich).

Morphometric analysis of testicular sections was performed using Motic DSAssistant software (Motic VM V1 Viewer 2.0, Motic Asia Corp., Kowloon, Hong Kong). Testicular damage and spermatogenesis were assessed histopathologically based on Johnson's mean testicular biopsy score (Johnson's score).^[^ ^66^, ^67^, ^68^ ^]^ For this analysis, three mice were used per group. Twenty‐five seminiferous tubules per mouse were evaluated for the presence or absence of germ cell types (spermatozoa, spermatids, spermatocytes, spermatogonia, Sertoli cells). Each tubule received a score from 1 to 10 based on these criteria. Morphometric analysis of seminiferous tubules was conducted at stages VII‐VIII of the seminiferous epithelium cycle to ensure consistent measurement conditions across all samples. Leydig cells were identified following the criteria described by Holm et al. (2003). Quantification was performed using histological sections from a minimum of three mice per group.^[^ ^69^ ^]^

### Apoptosis Analysis

TdT‐mediated dUTP‐biotin nick‐end labeling (TUNEL) staining was performed using a TUNEL kit (ab206386, Abcam, UK) according to manufacturer's instructions (n = 3 mice per group). The nuclei of apoptotic cells were stained brown, and apoptotic indices were calculated by selecting fifty fields randomly under a high‐power microscope (× 400). After staining, slides were washed three times with water and mounted with a coverslip using a xylene‐based DPX mounting medium (Sigma‐Aldrich). TUNEL‐positive cells (indicated by brown staining) were counted using Image J software (NIH, MD, USA). The level of apoptosis was evaluated by calculating the percentage of TUNEL‐positive cells relative to the total cell count.

### Testosterone Measurements

Serum testosterone levels were measured using R&D Systems Parameter Testosterone assay (KGE010) according to manufacturer's instructions. Plasma samples were diluted 1:10, and 100 µL was used per assay well (n = 4 mice per group).

### Sperm Count Analysis

The sperm concentration was determined using sperm extracted from the epididymal cauda. A 1:20 dilution was prepared by mixing epididymal cauda sperm with phosphate buffered saline. After thorough mixing, the diluted sample was loaded into a Neubauer counting chamber, and sperm cells were counted in five specific squares (four corners and center). The sperm concentration was calculated using the following formula:

Sperm Count (million/mL) = (Number of sperms in 5 squares of Neubauer's counting chamber) × 1,000,000

This calculation provided the sperm concentration in terms of million sperm per mL, with results adjusted according to the dilution factor.

### Microscopic video Analysis

Microscopic video recordings were performed using a Leica DM500 Microscope (Heerbrugg, Switzerland) equipped with a Leica ICC50 HD Microscope Camera (Heerbrugg, Switzerland) and controlled via the Leica LAS EZ software (Leica Microsystems). Videos were taken from representative samples of sperm using 10× and 40× objectives. A calibrated scale bar set to 30 µm, as shown on the software interface, was used for measurements. The samples were analyzed under brightfield settings. The calibration of the scale bar was conducted according to the manufacturer's guidelines to ensure accurate measurements during video acquisition.

### RNA Sequencing and Data Analysis

Total RNA was extracted from mouse testes (n = 4 per group) using RNeasy Micro Kit (Qiagen, Germany) according to manufacturer's protocol. The quality and quantity of the extracted RNA were assessed using a Tthestation (Agilent Technologies, USA) and a Qubit Flex Fluorometer (Thermo Fisher Scientific, USA), respectively. Only RNA samples with a quality value (RINe) of 7 or higher and a minimum quantity of 200 ng were selected for library preparation. To construct the sequencing library, rRNA was initially depleted using the rRNA Depletion Kit (MGI Tech, China). The RNA underwent fragmentation and reverse transcription with the RNA Directional Library Prep Kit (MGI Tech, China). The library for sequencing was finally completed using the High‐throughput (Rapid) Sequencing kit (MGI Tech, China). MGI DNBSEQ G400‐RS system was used for transcriptome sequencing at KNU NGS Core Facility (Daegu, South Korea). Following sequencing, reads were filtered using Trimmomatic to retain only those with a quality score of 30 or higher.^[^ ^70^ ^]^ More than 96% of paired reads from all samples survived, with detailed results shown in Table S4 (Supporting Information). The high‐quality reads were aligned to the reference genome (Mus_musculus.GRCm39.111) using HISAT2.^[^ ^71^ ^]^ Transcript assembly was subsequently performed using the alignment information with the StringTie2.^[^ ^72^ ^]^

### Statistical Analysis

Statistical analyses and visualization were conducted in RStudio (version 4.3.1) using the gene count matrix derived from prior analyses. Differentially expressed genes (DEGs) between two groups (CON vs PET) were identified using DESeq2, with criteria set at a p‐value of less than 0.05 and a log2 fold change greater than 2 or less than ‐2.^[^ ^73^ ^]^ Gene Ontology (GO) analysis of the DEGs was conducted using goseq, and the results were categorized into respective GO terms.^[^ ^74^ ^]^ Additionally, the Weighted Gene Co‐expression Network Analysis (WGCNA) was utilized to examine the relationships among gene expression patterns, facilitating the prediction of module clusters and analysis of their associations with phenotypic traits.^[^ ^75^ ^]^ Finally, genes exhibiting a topological overlap of 0.1 or higher within the selected module were extracted. Network analysis was then performed on eight reproduction‐related genes using Cytoscape (ver. 3.10.1).^[^ ^76^ ^]^ Kegg pathways were identified through pathview package in R studio.^[^ ^77^ ^]^ and for statistically significant genes, another statistical validation (Mann‐whitney test) was conducted by calculating TPM. The data presented in bar graphs were shown as mean ± SEM and analyzed using GraphPad 10.0 software (GraphPad, San Diego, USA). Statistical significance was tested using the unpaired *t*‐test for comparison between two groups.

## Conflict of Interest

The authors declare no conflict of interest.

## Author Contributions

S.J.J., G.D.L., and S.R.P. contributed equally to this work. The study was conceived and designed by B.M.R., S.J.L., and S.R.P. Experiments and animal study were conducted by S.R.P. and S.J.J. and data analyses were mainly done by G.D.L., S.J.J., and M.J.S. with contributions from some of the authors. The manuscript was written by S.J.J. and B.M.R. and finalized by B.M.R., with contributions from S.J.L., M.J.S and G.D.L. All authors discussed the results and have given approval to the final version of the paper.

## Supporting information



Supporting Information

Supplementary Video 1

Supplementary Video 2

Supplementary Video 3

Supplementary Video 4

Supplementary Video 5

Supplementary Video 6

## Data Availability

The data that support the findings of this study are available from the corresponding author upon reasonable request.
