# Supplementary material for: Unseen Threats: The Long‐term Impact of PET‐Microplastics on Development of Male Reproductive Over a Lifetime
Source: Adv Sci (Weinh). 2025 Jan 13;12(9):2407585. doi: 10.1002/advs.202407585 (PMC11884539; doi:10.1002/advs.202407585)
Supplement: Supplementary file 1 — Supporting Information [file ADVS-12-2407585-s007.docx]

Supporting Information for

**Unseen Threats: The Long-term Impact of PET-microplastics on Development of Male Reproductive over a Lifetime**

*Seungjin Jeong^1,2,^* †*, GyuDae Lee^3,^* †*, Surye Park^1,2,^* †*, Myeongjoo Son^4^, Seungjun Lee^1^*, Bomi Ryu^1,2^**

^1^ Department of Food Science and Nutrition, Pukyong National University, Busan, Republic of Korea

^2^ Department of Smart Green Technology Engineering, Pukyong National University, Busan, Republic of Korea

^3^ Department of Applied Biosciences, Kyungpook National University, Daegu, Republic of Korea
^4^ Department of Anatomy & Cell Biology, School of Medicine, Kangwon National University, Chuncheon, 24341, Republic of Korea

Seungjin Jeong, Surye Park, Seungjun Lee*, Bomi Ryu*

45, Yongso-ro, Nam-Gu, Busan, 48513, Republic of Korea.

E-mail: paul5280@pknu.ac.kr; bmryu@pknu.ac.kr

GyuDae Lee

80, Daehak-ro, Buk-gu, Daegu, 41566, Republic of Korea.

Myeongjoo Son

1, Kangwondaehakgil, Chuncheon-si, Gangwon state, 24341, Republic of Korea.

† Seungjin Jeong, GyuDae Lee, and Surye Park contributed equally to this work

* Corresponding author

Keyword: physically abraded PET-MPs, long-term ingestion, annual intake relevant dose, spermatogenesis, RNA transcriptome profiling


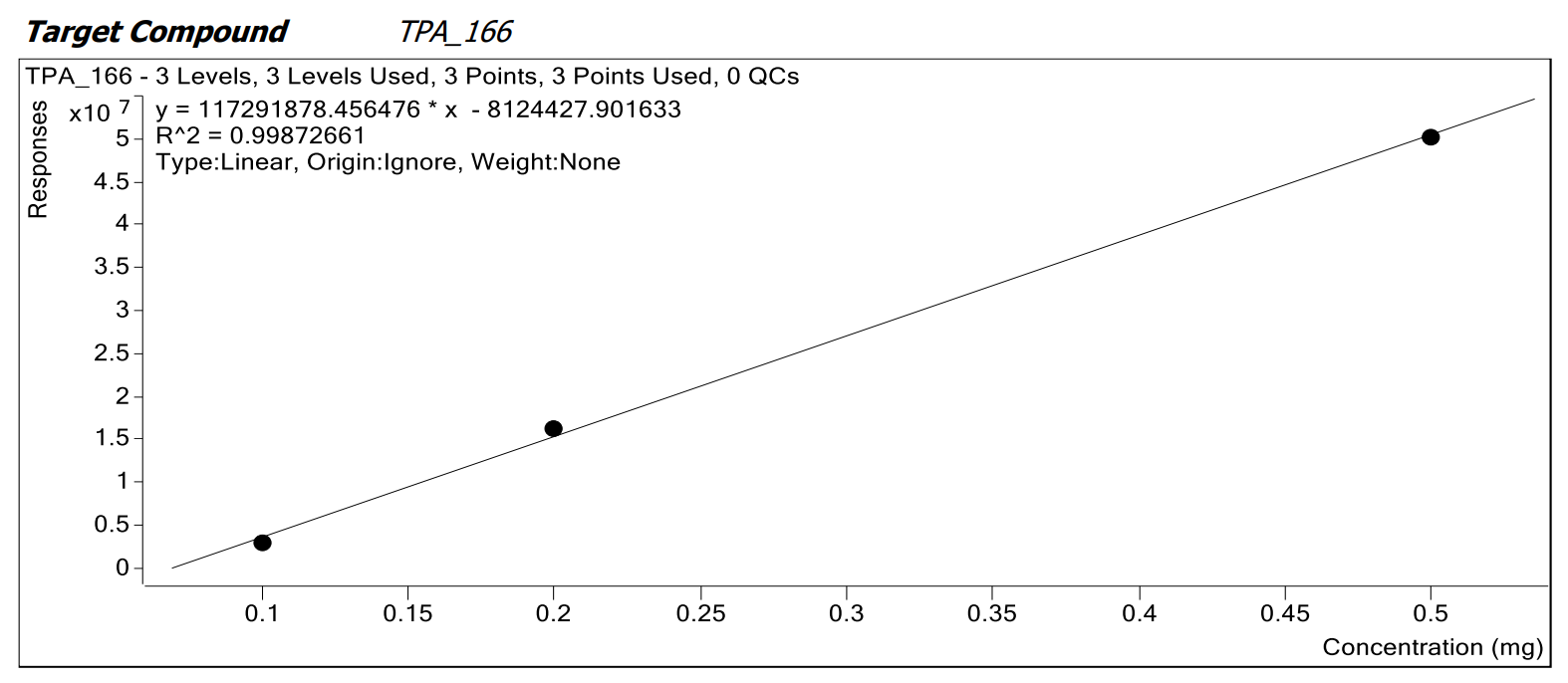


| **Target Compound** | **PET** | **TPA** | **Yield** |
| --- | --- | --- | --- |
| TPA_166 | 0.12 g | 0.1111 g | 92.58% |

**Figure S1. Calibration curve and yield of Terephthalic acid (TPA)**

The graph shows the linear relationship between the response and the concentration of TPA_166. The calibration was performed using three levels, with three points used. The response is measured in arbitrary units (x10^7^), and the concentration is measured in milligrams (mg). The yield of TPA_166. From 0.12 g of PET, 0.1111 g of TPA was obtained, resulting in a yield of 92.58%.


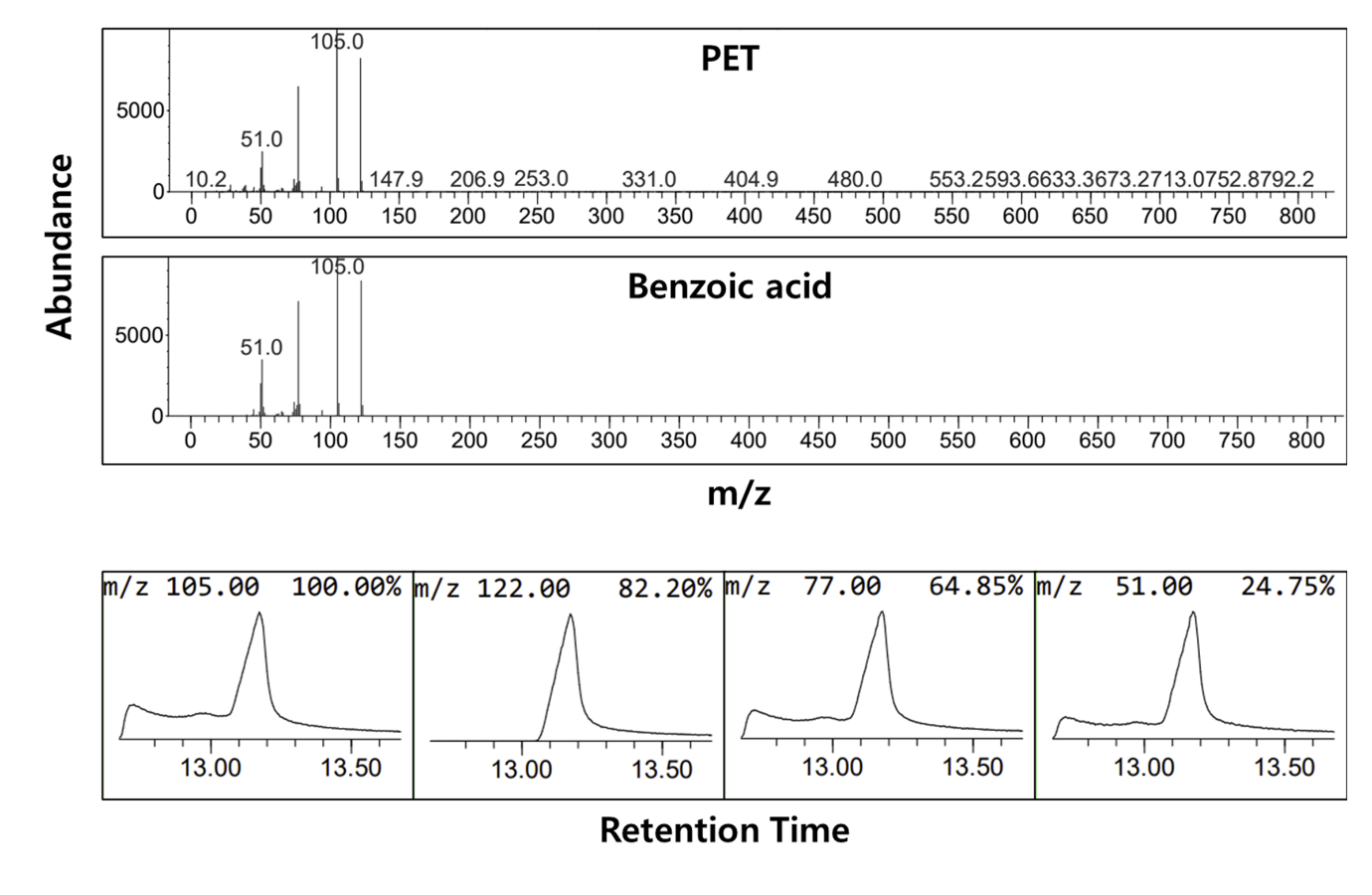


**Figure S2. Mass Spectra and Chromatographic Profiles of PET and Benzoic Acid**

(Top Panel) Mass Spectra of PET and Benzoic Acid: The top two spectra display the mass-to-charge (m/z) ratio versus abundance for PET and Benzoic Acid. The mass spectrum of PET (topmost spectrum) shows significant peaks at m/z values of 51.0, 105.0, 147.9, 206.9, and 253.0, among others. Similarly, the mass spectrum of Benzoic Acid (middle spectrum) also shows prominent peaks at m/z values of 51.0 and 105.0, indicating the presence of similar fragments in both substances.

(Bottom Panel) Chromatographic Profiles of Benzoic Acid: The bottom panel contains chromatograms for different m/z values (105.00, 122.00, 77.00, and 51.00) for Benzoic Acid. Each chromatogram displays the retention time in minutes versus the abundance. The retention times are consistent across different m/z values, all centered around 13.00 to 13.50 minutes, suggesting the detection of Benzoic Acid at these specific m/z values during the analysis. The percentage values (100.00%, 82.20%, 64.85%, and 24.75%) indicate the relative abundance of these ions in the sample.

Overall, this figure provides a comparative analysis of the mass spectra of PET and Benzoic Acid, along with the chromatographic profiles of Benzoic Acid at specific m/z values.


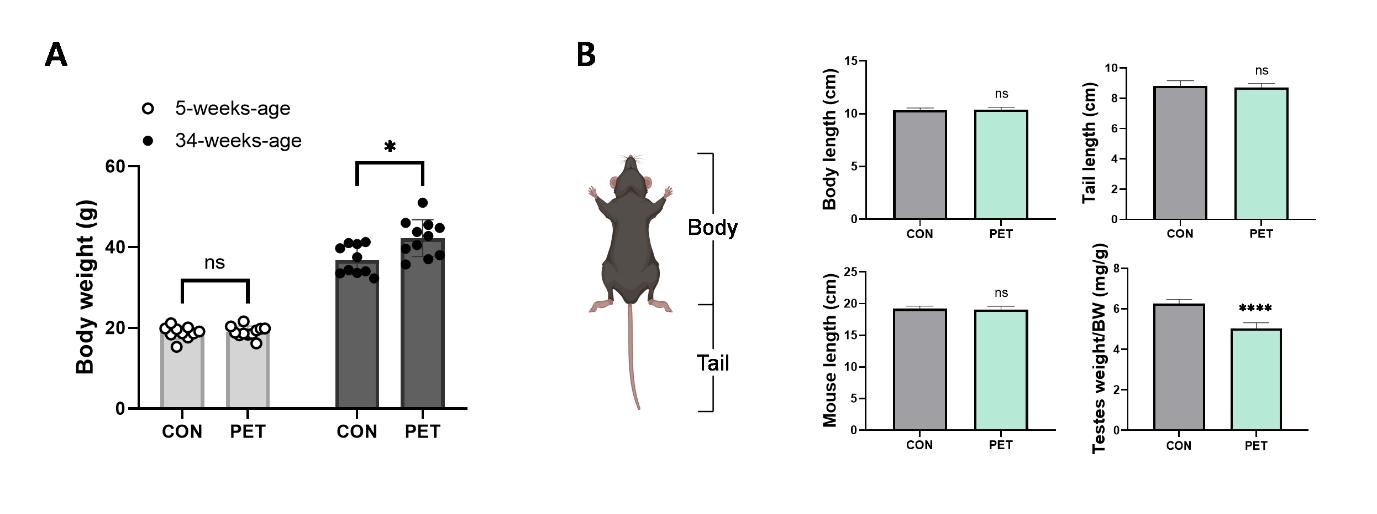
 **Figure S3. Effects of PET-MPs ingestion on body and testes metrics in mice.**

(A) Body weight comparison between CON and PET at two different ages: 5 weeks and 34 weeks. Open circles represent 5-week-old mice, while filled circles represent 34-week-old mice.

(B) Results of body length, tail length, total mouse length, and testes weight relative to body weight (testes weight/BW) in CON and PET.

The data are presented as the means ± SDs. ****p < 0.0001; n.s., p > 0.05. Statistical analysis in panels B was performed by unpaired Student's t-test


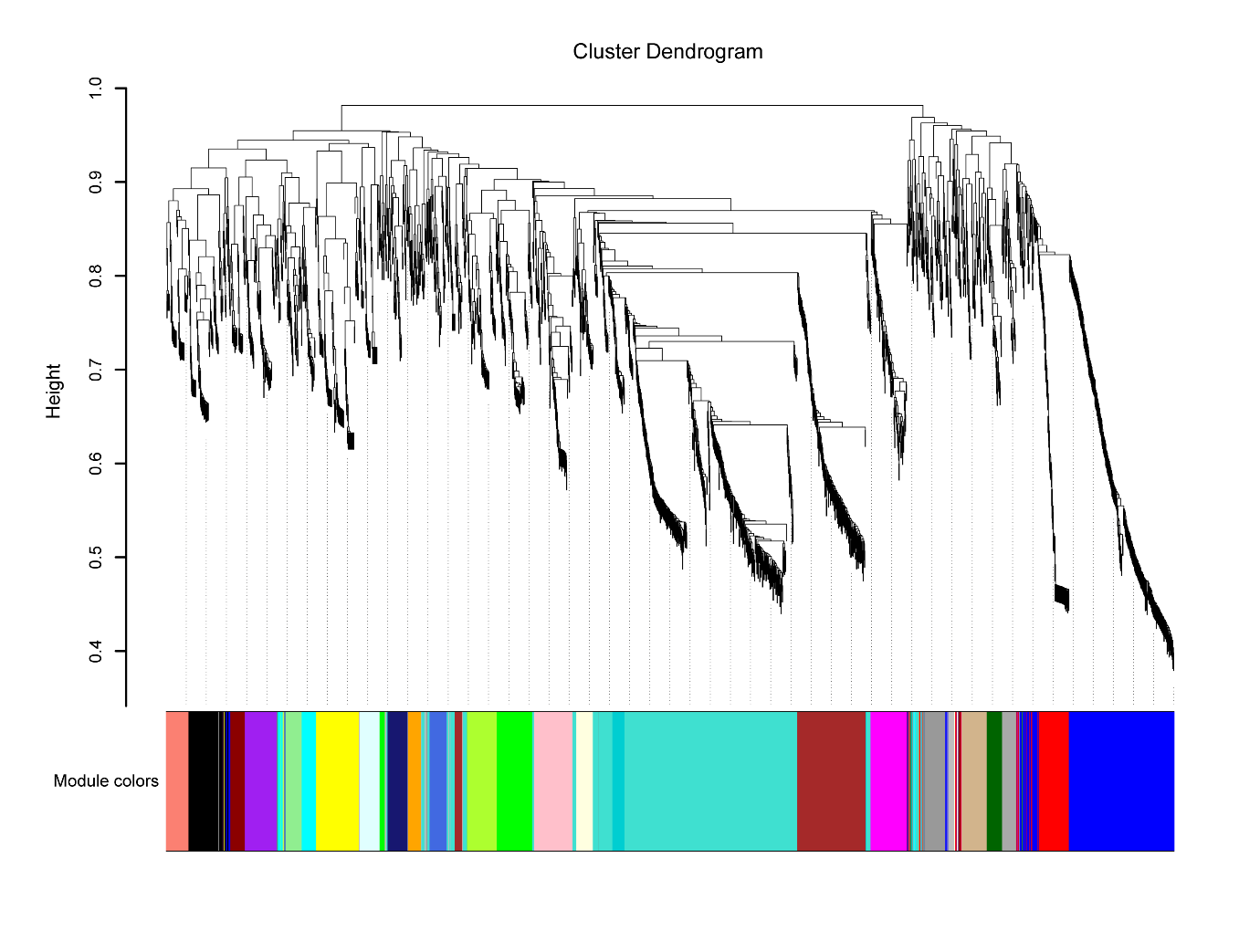


**Figure S4. Dendrogram generated through average linkage hierarchical clustering of gene expression data.** The colored bar beneath the dendrogram indicates module assignments as determined by the Dynamic Tree Cut method.

**
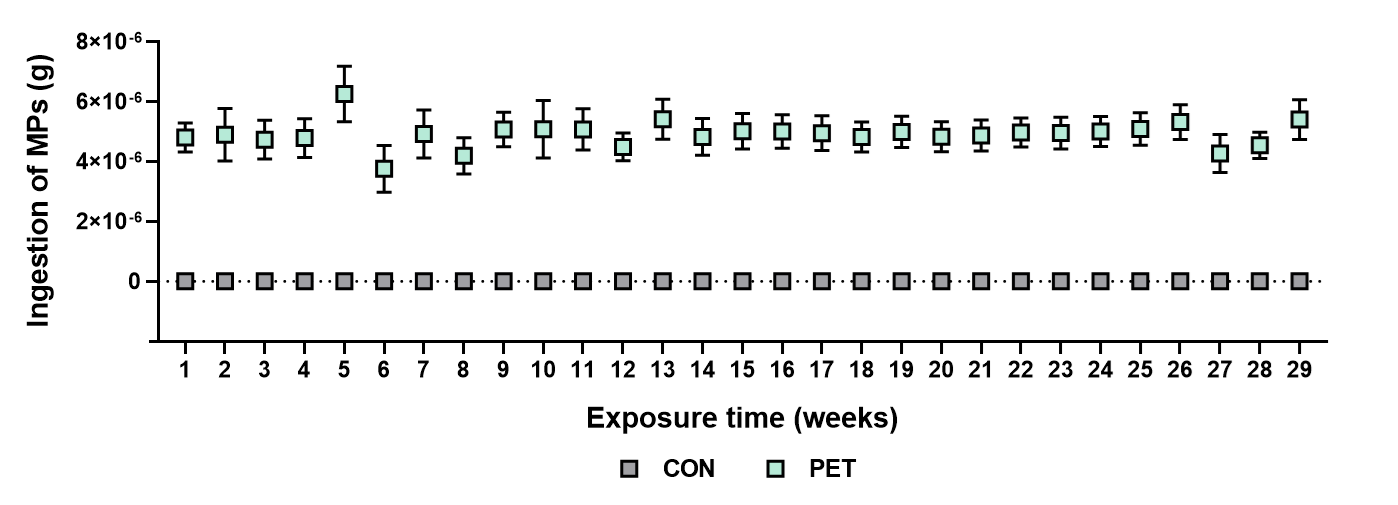
**

**Figure S5. Weekly PET-MP intake throughout the exposure period is shown in the graph, which represents the calculated exposure levels of PET-MPs in the feed.** The levels were determined by calculating the difference between the initial amount provided and the residual feed collected and weighed after feeding. The graph shows the weekly intake of PET-MPs in CON and PET over 29 weeks of exposure. Data are presented as mean ± SEM.

**Table S1. Compounds Identified in Py-GC-MS results of PET-MPs**

| **Compound number (#)** | **RT (min)** | **Area (%)** | **Hit Name** | **Quality** | **m/z** | **CAS Number** |
| --- | --- | --- | --- | --- | --- | --- |
| 1 | 1.85 | 18.77 | Carbon dioxide | 4 | 44, 28, 26, 27.1, 16 | 000124-38-9 |
| 2 | 2.014 | 5.93 | Acetaldehyde | 91 | 44, 29, 43, 28, 18 | 000075-07-0 |
| 3 | 4.082 | 0.51 | Benzene | 94 | 78, 28, 77, 52, 32 | 000071-43-2 |
| 4 | 12.724 | 2.3 | 1,2-Propanedione, 1-phenyl- | 87 | 105, 77, 28, 51, 106 | 000579-07-7 |
| 5 | 12.962 | 0.47 | Vinyl benzoate | 86 | 105, 77, 28, 51, 106 | 000769-78-8 |
| 6 | 13.177 | 12.48 | Benzoic acid | 96 | 105, 122, 77, 51, 50 | 000065-85-0 |
| 7 | 14.545 | 0.87 | Thioxan-3-one, oxime | 83 | 131, 77, 105, 103, 28 | 058230-51-6 |
| 8 | 14.65 | 1.69 | Naphthalene, 2-ethenyl- | 93 | 154, 153, 152, 77, 105 | 000827-54-3 |
| 9 | 15.473 | 0.98 | Benzimidazole, 2-amino-1-methyl- | 72 | 147, 105, 77, 28, 122 | 001622-57-7 |
| 10 | 15.721 | 12.78 | 2-Propenal, 3-[4-(dimethylamino)phenyl]- | 47 | 175, 104, 76, 132, 147 | 006203-18-5 |
| 11 | 15.894 | 0.11 | Benzoic acid, 2-formyl- | 38 | 105, 175, 122, 77, 28 | 000119-67-5 |
| 12 | 16.004 | 0.3 | Methanol, oxo-, benzoate | 38 | 105, 177, 122, 77, 28 | 1000305-65-2 |
| 13 | 16.05 | 1.94 | Benzoic acid, 2-(1-oxopropyl)- | 62 | 149, 105, 122, 77, 65 | 002360-45-4 |
| 14 | 16.164 | 0.47 | 1,3-Dioxolane, 2-methyl-2-phenyl- | 53 | 149, 105, 122,77, 121 | 003674-77-9 |
| 15 | 16.242 | 1.49 | Benzoic acid, 2-(1-oxopropyl)- | 53 | 149, 105, 77, 65, 121 | 002360-45-4 |
| 16 | 16.343 | 0.51 | 4-Acetylbenzoic acid | 59 | 149, 105, 65, 121, 77 | 000586-89-0 |
| 17 | 17.258 | 0.54 | 1,2-Benzenedicarboxylic acid, bis(1-methylethyl) ester | 72 | 149, 121, 65, 28, 105 | 000605-45-8 |
| 18 | 17.358 | 0.63 | 1,2-Benzenedicarboxylic acid, monobutyl ester | 72 | 149, 121, 65, 28, 150 | 000131-70-4 |
| 19 | 17.459 | 0.38 | Terephthalic monohydroxamic acid | 80 | 149, 65, 121, 28, 150 | 022372-40-3 |
| 20 | 17.537 | 0.86 | Benzoic acid, 2-(1-oxopropyl)- | 68 | 149, 121, 65, 193, 219 | 002360-45-4 |
| 21 | 17.624 | 1.75 | Fenbufen | 49 | 149, 181, 152, 153, 121 | 036330-85-5 |
| 22 | 17.743 | 0.86 | Benzene, 1-isothiocyanato-2-methyl- | 64 | 149, 65, 121, 28, 73 | 000614-69-7 |
| 23 | 18.859 | 2.86 | Diethylene glycol dibenzoate | 53 | 105, 77, 149, 106, 51 | 000120-55-8 |
| 24 | 19.142 | 0.33 | p-Terphenyl | 93 | 149, 230.1, 207, 28, 105 | 000092-94-4 |
| 25 | 19.28 | 0.63 | Tetracosane | 93 | 149, 57.1, 71.1, 207, 43.1 | 000646-31-1 |
| 26 | 19.728 | 1.01 | Tetracosane | 98 | 57.1, 71.1, 43.1, 85.1, 149 | 000646-31-1 |
| 27 | 19.911 | 0.28 | 3-Methyl-2,3-dihydro-benzo[b]thiophene-3-carboxylic acid, methyl ester | 18 | 149, 207, 133, 105, 28 | 039891-64-0 |
| 28 | 20.021 | 1.52 | Pentylone, N-TFA | 25 | 251, 207, 149, 131, 105 | 1000445-92-5 |
| 29 | 20.19 | 1.3 | Pentacosane | 99 | 57.1, 71.1, 43.1, 85.1, 207 | 000629-99-2 |
| 30 | 20.254 | 0.29 | Benzo[h]quinoline, 2,4-dimethyl- | 46 | 207, 149, 28, 281, 105 | 000605-67-4 |
| 31 | 20.67 | 1.47 | Eicosane | 99 | 57.1, 71.1, 85.1, 43.1, 207 | 000112-95-8 |
| 32 | 20.826 | 0.39 | Terephthalic acid, di(2-methoxyethyl) ester | 35 | 207, 105, 149, 281, 147 | 1000323-88-7 |
| 33 | 20.986 | 10.31 | 2H-pyrrol-2-one, 1,5-dihydro-4-hydroxy-3-[(4-methylphenyl)thio]-1-phenyl- | 45 | 297.1, 149, 105, 298.1, 104 | 1000397-44-9 |
| 34 | 21.183 | 1.48 | Eicosane | 97 | 57.1, 71.1, 43.1, 85.1, 207 | 000112-95-8 |
| 35 | 21.746 | 1.14 | Tetracosane | 97 | 57.1, 71.1, 207, 85.1, 43.1 | 000646-31-1 |
| 36 | 22.253 | 0.43 | Tricyclo[4.2.1.0(2,5)]non-7-ene, 3,4-di(tris(trimethylsilyloxy)silyl)- | 49 | 207, 325.1, 281, 28, 177 | 1000381-62-9 |
| 37 | 22.363 | 0.65 | Silicic acid, diethyl bis(trimethylsilyl) ester | 38 | 207, 57.1, 71.1, 43, 85.1 | 003555-45-1 |
| 38 | 22.455 | 0.66 | Acetamide, N-[4-[2-[(3-methylphenyl)amino]-4-thiazolyl]phenyl]- | 30 | 207, 323.1, 281, 175, 28 | 1000319-57-6 |
| 39 | 23.063 | 0.37 | 2'-Hydroxy-5'-methylacetophenone, TMS derivative | 47 | 207, 281, 28, 57.1, 71.1 | 097389-69-0 |
| 40 | 23.704 | 0.74 | Silicic acid, diethyl bis(trimethylsilyl) ester | 37 | 207, 281, 28, 105, 149 | 003555-45-1 |
| 41 | 23.946 | 7.53 | Benzo[c]phenanthridin-6(5H)-one, 11,12-dihydro-2,3,8,9-tetramethoxy | 49 | 367.1, 104, 325, 368.1, 162 | 1000115-69-5 |

**Table S2. Johnson's Score and Corresponding Histological Criteria for Spermatogenesis**

| **Johnson's Score** | **Histological criteria** |
| --- | --- |
| 10 | Full spermatogenesis |
| 9 | Slightly impaired spermatogenesis, many late spermatids, disorganized epithelium |
| 8 | Less than five spermatozoa per tubule, few late spermatids |
| 7 | No spermatozoa, no late spermatids, many early spermatids |
| 6 | No spermatozoa, no late spermatids, few early spermatids |
| 5 | No spermatozoa or spermatids, many spermatocytes |
| 4 | No spermatozoa or spermatids, few spermatocytes |
| 3 | Spermatogonia only |
| 2 | No germinal cells, Sertoli cells only |
| 1 | No seminiferous epithelium |

**Table S3. Expressed Genes in RNA-Seq Module Analysis between the CON and PET**

| **No** | **Gene** | **Summary** |
| --- | --- | --- |
| 1 | *Kash5* | Enables dynein complex binding activity and identical protein binding activity. Involved in cytoskeleton organization; spindle localization; and telomere localization. Acts upstream of or within double-strand break repair via homologous recombination; gamete generation; and homologous chromosome segregation. Located in chromosome; meiotic spindle pole; and nuclear outer membrane. Part of meiotic nuclear membrane microtubule tethering complex. Is expressed in spermatocyte. Orthologous to human KASH5 (KASH domain containing 5). [provided by Alliance of Genome Resources, Apr 2022] |
| 2 | *Meiosin* | Predicted to enable DNA binding activity and protein dimerization activity. Involved in several processes, including activation of meiosis; cellular response to retinoic acid; and gamete generation. Located in nucleus. Is expressed in genitourinary system. Orthologous to human MEIOSIN (meiosis initiator). [provided by Alliance of Genome Resources, Apr 2022] |
| 3 | *Alkbh5* | Enables mRNA N6-methyladenosine dioxygenase activity. Involved in oxidative single-stranded RNA demethylation; regulation of mRNA stability; and spermatogenesis. Located in nuclear speck. Is expressed in brain. Orthologous to human ALKBH5 (alkB homolog 5, RNA demethylase). [provided by Alliance of Genome Resources, Apr 2022] |
| 4 | *Ace* | Enables metallopeptidase activity and peptidyl-dipeptidase activity. Involved in several processes, including cell proliferation in bone marrow; positive regulation of protein modification process; and regulation of gene expression. Acts upstream of or within several processes, including heart contraction; neutrophil mediated immunity; and positive regulation of systemic arterial blood pressure. Located in cytoplasm and plasma membrane. Is expressed in several structures, including alimentary system; brain; extraembryonic component; genitourinary system; and integumental system. Human ortholog(s) of this gene implicated in several diseases, including artery disease (multiple); autoimmune disease (multiple); gastrointestinal system cancer (multiple); heart valve disease (multiple); and lung disease (multiple). Orthologous to human ACE (angiotensin I converting enzyme). [provided by Alliance of Genome Resources, Apr 2022] |
| 5 | *Arid4a* | Predicted to enable transcription cis-regulatory region binding activity. Acts upstream of or within several processes, including establishment of Sertoli cell barrier; peptidyl-lysine trimethylation; and regulation of gene expression. Predicted to be located in cytosol; nucleoplasm; and plasma membrane. Predicted to be part of transcription repressor complex. Predicted to be active in nucleus. Used to study acute myeloid leukemia. Orthologous to human ARID4A (AT-rich interaction domain 4A). [provided by Alliance of Genome Resources, Apr 2022] |
| 6 | *Akap9* | Predicted to enable several functions, including potassium channel regulator activity; protein kinase A regulatory subunit binding activity; and transmembrane transporter binding activity. Acts upstream of or within Sertoli cell development and spermatogenesis. Located in several cellular components, including 9+2 motile cilium; microtubule organizing center; and subapical part of cell. Is expressed in central nervous system; pancreas epithelium; and retina. Human ortholog(s) of this gene implicated in long QT syndrome 11. Orthologous to human AKAP9 (A-kinase anchoring protein 9). [provided by Alliance of Genome Resources, Apr 2022] |
| 7 | *Golga3* | Predicted to act upstream of or within cell differentiation and spermatogenesis. Located in Golgi membrane and endoplasmic reticulum-Golgi intermediate compartment. Is expressed in several structures, including cardiovascular system; endocrine gland; genitourinary system; gut; and nervous system. Orthologous to human GOLGA3 (golgin A3). [provided by Alliance of Genome Resources, Apr 2022] |
| 8 | *Wipf3* | Predicted to enable SH3 domain binding activity. Predicted to be involved in actin filament-based movement. Predicted to act upstream of or within cell differentiation and spermatogenesis. Predicted to be located in cytoplasm. Predicted to be active in actin filament. Orthologous to human WIPF3 (WAS/WASL interacting protein family member 3). [provided by Alliance of Genome Resources, Apr 2022] |
| 9 | *Herc2* | Predicted to enable SUMO binding activity; ubiquitin protein ligase activity; and ubiquitin protein ligase binding activity. Involved in proteasome-mediated ubiquitin-dependent protein catabolic process. Acts upstream of or within spermatogenesis. Located in mitochondrial inner membrane. Is expressed in several structures, including central nervous system; ganglia; and trunk. Human ortholog(s) of this gene implicated in autosomal recessive non-syndromic intellectual disability and pigmentation disease. Orthologous to human HERC2 (HECT and RLD domain containing E3 ubiquitin protein ligase 2). [provided by Alliance of Genome Resources, Apr 2022] |

**Table S4. Sequencing quality filtering results**

| **sample_ID** | **Both survived** | **Forward only** | **Reverse only** | **Dropped** | **group** |
| --- | --- | --- | --- | --- | --- |
| CON1 | 29408609 (97.70%) | 422594 (1.40%) | 224799 (0.75%) | 44071 (0.15%) | control |
| CON2 | 27177217 (96.99%) | 542565 (1.94%) | 245514 (0.88%) | 55465 (0.20%) | control |
| CON3 | 28345123 (96.98%) | 567040 (1.94%) | 261337 (0.89%) | 55635 (0.19%) | control |
| CON4 | 28953796 (97.10%) | 557225 (1.87%) | 250072 (0.84%) | 56383 (0.19%) | control |
| PET1 | 25498350 (97.15%) | 484742 (1.85%) | 216007 (0.82%) | 48276 (0.18%) | PET |
| PET2 | 29831281 (96.95%) | 560910 (1.82%) | 313975 (1.02%) | 62701 (0.20%) | PET |
| PET3 | 33576103 (97.03%) | 678534 (1.96%) | 285417 (0.82%) | 64743 (0.19%) | PET |
| PET4 | 27055962 (98.03%) | 322292 (1.17%) | 190213 (0.69%) | 31260 (0.11%) | PET |

**Video S1. Comparison of Sperm Motility in CON and PET**
